# Mark–Release–Recapture (MRR) of Sterile Male *Aedes albopictus* (Skuse) in Sri Lanka: Field Performance of Sterile Males and Estimation of the Wild Mosquito Population Density

**DOI:** 10.3390/insects15070466

**Published:** 2024-06-22

**Authors:** Menaka Hapugoda, Nilmini Silva Gunawardena, Tharaka Ranathunge, Jeremy Bouyer, Hamidou Maiga, Kankanige Karunathilake, Gayan Parakrama Withanage, Indika Weerasinghe, Bazoumana B. D. Sow, Jeevanie Harishchandra

**Affiliations:** 1 Molecular Medicine Unit, Faculty of Medicine, University of Kelaniya, Ragama 11010, Sri Lanka; nilminisg@kln.ac.lk (N.S.G.); gayan.parakrama@gmail.com (G.P.W.); 2 Department of Zoology, Faculty of Science, Eastern University, Batticaloa 30000, Sri Lanka; ranathungermtb@esn.ac.lk; 3 Insect Pest Control Subprogramme, Joint FAO/IAEA Centre of Nuclear Techniques in Food and Agriculture, Department of Nuclear Sciences and Applications, International Atomic Energy Agency, 1400 Vienna, Austria; jeremy.bouyer@cirad.fr (J.B.); maigahamid@yahoo.fr (H.M.); 4 UMR ASTRE (Animal Santé Territoires Risques et Ecosystèmes), CIRAD, Plate Forme CYROI, 2 rue Maxime Rivière, 97491 Sainte-Clotilde, La Réunion, France; 5 Institut de Recherche en Sciences de la Santé (IRSS), Bobo-Dioulasso 01 BP 545, Burkina Faso; sowbazoumana@gmail.com; 6 Department of Sociology, Faculty of Social Science, University of Kelaniya, Kelaniya 11010, Sri Lanka; kkarunathilake@gmail.com; 7 National Dengue Control Unit, Public Health Complex, Ministry of Health, Narahenpita, Colombo 01000, Sri Lanka; indika4weerasinghe@gmail.com; 8 Anti-Malaria Campaign (AMC), Public Health Complex, Ministry of Health, Narahenpita, Colombo 01000, Sri Lanka; jeevanieharishchandra@yahoo.com

**Keywords:** dengue, sterile male mosquitoes, sterile insect technique

## Abstract

**Simple Summary:**

Dengue is an endemic disease in Sri Lanka causing frequent cyclical epidemics. *Aedes albopictus* is a dengue vector mosquito which is widely spread in many parts of the country. The Sterile Insect Technique (SIT) is a novel dengue vector control approach to be integrated with the currently available vector control methods. A series of Mark–Release–Recapture (MRR) experiments were conducted in Sri Lanka against *Ae. albopictus* using gamma-ray-sterilized males to evaluate the field performance of the sterilized males and estimate the wild mosquito population size to be used for future SIT trials. Results obtained from this study can be used for planning future pilot field trials in Sri Lanka.

**Abstract:**

Dengue is an important mosquito-borne disease in Sri Lanka. The Sterile Insect Technique (SIT) is an environment-friendly and novel method that can suppress dengue vector mosquitoes in Sri Lanka. This study aimed to evaluate the field performance of sterile males and the density of wild male *Aedes albopictus* (Skuse) using a Mark–Release–Recapture (MRR) assay. Laboratory-colonized male pupae were exposed to 50 Gy gamma using a Co^60^ source. Sterile males (approx. 10,000) marked with fluorescent dust were released weekly for 4 consecutive weeks (January–February 2021) in a geographically isolated 30 ha site in Gampaha. Results show sterile males could disperse up to 543.8 m with a mean distance of 255.1 ± 44.6 m and survive up to 6 days with a mean life expectancy of 3.55 ± 2.32 days. A high field mating competitiveness of sterile males based on a Fried value of 0.47 ± 0.007 and significant induced sterility in the wild eggs in the second generation were found. The mean wild male mosquito population density was 163 males/ha. The data generated will be useful for designing future trials in Sri Lanka and other countries with similar situations.

## 1. Introduction

The Asian tiger mosquito *Aedes albopictus* (Skuse, 1894) is an invasive species responsible for the transmission of arboviruses such as dengue, Zika, and chikungunya [1]. This species, native to Southeast Asia, has colonized all continents [2]. In the absence of efficient vaccines to prevent these diseases, vector control remains a key strategy. The Sterile Insect Technique (SIT) is presented as a very promising and environment-friendly control tool [1] used to control insect pests by releasing a large number of sterile males into the wild population. These sterile males will compete with wild fertile males to mate with females in the field and thereby reduce the fertility of the target population [3,4]. The SIT relies on the rearing, sterilization, and release of male mosquitoes in sufficient numbers to have an impact on populations when they mate with wild females in the field, producing nonviable eggs [5]. Further, SIT does not generate resistance, and the released sterile males can disperse and access sites where virgin females are found [6]. The SIT is increasingly gaining momentum worldwide as a component of area-wide-integrated pest management [7] and was tested to suppress *Ae. albopictus* in a number of countries, including Italy [8,9,10], Mauritius [11], Reunion Island [12,13], Greece [14,15], Germany [16], and Spain [1], as well as *Ae. aegypti* in Cuba [17].

Knowledge of the characteristics of sterile males and reliable quantification of wild population density are prerequisites for planning SIT interventions [18,19]. Mark–Release–Recapture (MRR) protocols can be used to evaluate the above-mentioned parameters, including sterile male dispersal capacity, survival rate, and their field competitiveness in an open field setting, as well as the density of the wild population [20,21]. Dispersal in mosquitoes has been investigated using MRR designs in which adult mosquitoes are marked with dusts, dyes, paints, trace elements, and radioactive isotopes [22]. Fluorescent dust can be used for MRR studies [7,20,21,23]. Typically, these marked adult individuals are released at a specific point and then subsequently recaptured at other sites [24].

Before the field application of SIT (phase II), the field performance of sterile male mosquitoes and the density of wild male mosquitoes need to be assessed (phase I), as recommended in the phased conditional approach for testing the SIT against *Aedes* mosquitoes [18,25]. MRR has been conducted in several countries for *Ae. albopictus* in Mauritius [26,27], Reunion Island [28], Indonesia [29], Italy [23], and Albania [7], as well as *Ae. aegypti* in Brazil [30] and Captiva Island, USA [31].

Dengue is the most important mosquito-borne viral infection, causing enormous social and economic burden in Sri Lanka [32]. *Ae. albopictus* is one of the most important and widely spread dengue vector mosquitoes in Sri Lanka. The feasibility of integrating SIT into the Integrated Vector Management (IVM) in Sri Lanka needs to be considered.

In this study, we assessed (1) the dispersal, survival, and mating competitiveness of a radio-sterilized local strain of *Ae. albopictus*, (2) the density of wild *Ae. albopictus* male mosquitoes, and (3) the sterile-to-wild male ratio using an MRR assay. 

## 2. Materials and Methods

### 2.1. Mosquito Rearing and Irradiation 

A local strain of *Ae. albopictus* was colonized from wild eggs collected from the release site. The mosquito colony was maintained in 30 × 30 × 30 cm mosquito-rearing metal cages (John W. Hock Company, Gainesville, FL, USA), under a 12:12 h (light:dark) cycle at standard conditions (at 27 ± 2 °C and 75 ± 5% humidity) in a confined insectary at the Molecular Medicine Unit, Faculty of Medicine, University of Kelaniya, Ragama, Sri Lanka [33], and the 2nd generation of mosquitoes was used for MRR experiments. Male *Ae. albopictus* pupae were separated using a Fay–Morlan glass plate sorter (M5412, John W. Hock Company, Gainesville, FL, USA) [34,35].

Male pupae (1000) aged 24 h were transferred with 300 mL of water into pupal cups of 1 L volume and transported at 25 °C to the irradiation facility located in the Horticultural Crop Research and Development Institute, Peradeniya, Sri Lanka. The water volume was reduced from the pupal cup, and pupae were transferred into petri plates with 15 mL of water. Six petri plates were inserted into the irradiating chamber and exposed to a predetermined optimal dose of 50 Gy (Gamma rays) for 1 min [36,37] using an irradiator (Gammacell 220, Atomic Energy of Canada Ltd., Chalk River, ON, Canada, Co^60^). Pupae were transferred into the cup, and the water level was increased to 500 mL and the pupae were brought back to the laboratory. Pupal bowls were placed into metallic mosquito cages (30 cm × 30 cm × 30 cm) under standard laboratory conditions [at 27 ± 2 °C, 75 ± 5% relative humidity and a photoperiod of 12:12 h (L:D) with 10% sucrose solution] for 2 days until all pupae became adults.

### 2.2. Sterile Male Mosquito Marking

Sterile male mosquitoes aged 2 days were fed with 10% sucrose solution and maintained in the same metallic cages (approx. 2500/cage) one day before release. Two cages with approx. 5000 males were marked with fluorescent dust (RADGLO^®^ JST, Radiant NV, Houthalen, Belgium or DayGlo^®^ Color Corp., Cleveland, OH, USA) (750 mg/2500 adults) and dispersed through the net [20]. Another set of two cages with 5000 sterile males was marked using a different color. Every week, 10,000 sterile male mosquitoes marked with two colors to differentiate release site and time were prepared to release at two different release points. The same colors were repeated to mark mosquitoes after 2 weeks (Supplementary Table S1).

### 2.3. Selection of Mosquito Release and Control Sites 

Two semiurban residential areas located in the District of Gampaha, Kidagammulla Grama Niladhari Division (GN), with 30 ha area (6°54′5″ and 7°20′) and Aluthgama, GN Division, with 30 ha rectangular area (79°4′75″ and 80°13′) were selected as release and control sites, respectively. The release site, a geographically isolated semiurban area is a representative of the future pilot SIT field trial site. The control site, which is located 1260 km apart from the release site, had similar geographical and environmental characteristics. 

### 2.4. Selection of Mosquito Release Points and Entomological Monitoring Stations 

Two mosquito release points set 200 m apart were selected in the release site, and 50 m apart radius circles around each release point (50 m, 100 m, 150 m, and 200 m) were marked on the Geographical Information System (GIS) map of the release site following the Insect Pest Control Laboratory guidelines [20].

In the release site, a total of 84 entomological monitoring stations (at least 2 per ha), including 20 Bio Gene (BG) sentinel traps and 8 Human Landing Collection (HLC) sites to trap adult mosquitoes together with 56 ovitraps, were placed at different locations in the concentric rings. At the same time, 34 entomological monitoring stations with 10 BG traps, 4 HLC sites, and 20 ovitraps were also placed in the control site in concentric rings.

**Figure 1:**
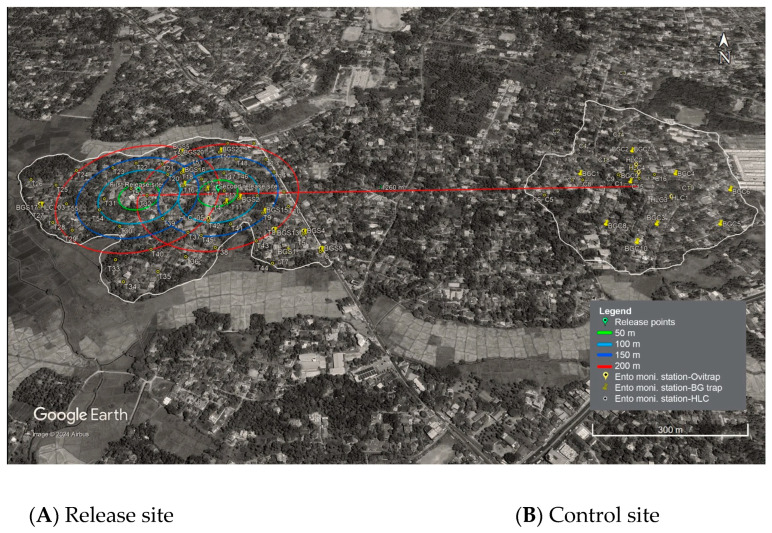
Map of MRR release and control sites in Gampaha District, Sri Lanka. Concentric lines represent four annuli at 50, 100, 150, and 200 from two release points in the release site: (**A**) Release site with 2 release points and entomological trapping stations (20 BG traps, 8 HLC sites, and 56 ovitraps. (**B**) Control site with entomological trapping stations for (10 BG traps, 4 HLC sites, and 20 ovitraps).

The location of each entomological monitoring station (Supplementary Material Annex 1) was incorporated into the map of each site. The map was prepared using Google Earth Pro [version 7.3.6.9796 (64-bit)] using the default WGS84 default georeferencing settings (Figure 1).

### 2.5. Community Awareness 

A community awareness campaign for releasing marked sterile male mosquitoes was conducted by a team including a sociologist and two community mobilizers, who highlighted the safety of the release of sterile male mosquitoes. In this effort, the social mobilization team conducted Focus Group Discussions (FGDs) and community awareness meetings to convince and inform inhabitants, government officers, and community leaders of the area about the study. 

### 2.6. Mosquito Releases 

Marked sterile male mosquitoes (approx. 10,000) were transported to the release site at ambient temperature. Marked mosquitoes with one color (approx. 5000) were released at one release point. Releases were conducted early in the morning to avoid mortality and clumping of the mosquitoes. Releases were achieved once a week for four consecutive weeks (22 January 2021–12 February 2022). The males that remained in the cage after 30 min were considered dead and not considered in the statistical analysis.

Mosquito releases were conducted for 4 weeks, with approx. 10,000 sterile males for each release site/time divided into two release points with different colors (pink, blue, white, and yellow) (Supplementary Table S1). 

### 2.7. Entomological Monitoring

(i) Adult mosquito recapture at trapping stations and identification

BG-sentinel traps baited with standard BG-lure were operated from the date of release up to 12 days after release (22 January 2021–24 February 2021). Adult mosquitoes were recaptured using BG traps and HLC for adults (every other day) at trapping points at release and control sites. Marked and unmarked mosquitoes were identified using a handheld Ultraviolet (UV) lamp (UVP, CA, USA). Distance from the release point to the sampling station (dispersal) was measured using GPS coordinates.

(ii) Mosquito egg collection and hatching

Ovitraps consisted of 400 mL black plastic containers holding 150 mL of dechlorinated water and an egg germination paper placed at the release (10) and control (20) sites for collecting eggs. The collected eggs were used to estimate the induced sterility and the natural fertility, respectively, during MRR. Ovitraps were operated from the date of release up to 14 days after release (22 January 2021–26 February 2021). Mosquito population dynamics were monitored in the release and control areas by counting and hatching the eggs collected in the ovitraps monitoring system. Egg papers were collected from ovitraps weekly, transferred to the laboratory in polythene bags (not completely sealed), and transported at ambient temperature. The number of eggs on each paper was counted under a stereomicroscope the following day and hatched at least 14 days after the collection day to assess their fertility rate in the release areas in comparison with the control area [7]. 

### 2.8. Collection of Weather Parameters

Weather parameters (mean temperature, relative humidity, and rainfall) were collected from the Meteorological Department, Colombo 07, Sri Lanka.

### 2.9. Ethical Aspects

Ethical permission for this study was obtained from the Ethical Review Committee, Institute of Biology, Sri Lanka (IOBSL 207 02 2020).

### 2.10. Data Management and Statistical Analysis

Data collection involved paper-based methods in both field and laboratory settings. Upon collection, data were transcribed into Microsoft Excel 2016 for initial organization. To ensure compatibility with R software 4.1.1, date, decimal, and variable name formatting were adjusted according to the R convention [38]. A double-entry system was implemented for accuracy, with discrepancies resolved through verification.

The dataset was imported into R software version 4.1.1 [39] for statistical analysis after preprocessing. MRR results were then analyzed using the ‘sit-r’ package (version 1.1.2.9013) [40]. We employed a generalized linear model with a Poisson distribution and log link function to assess potential differences in key parameters associated with trapping methods, marking colors, and release time. To assess the variation in natural egg fertility between release and control sites, we employed a binomial Generalized Linear Mixed Model (GLMM) with maximum likelihood estimation via Laplace Approximation. Egg hatch served as the response variable, and an ovitrap station was incorporated as a random effect to account for potential clustering within sites. We investigated male dispersal patterns by analyzing three key metrics: Mean Distance Traveled (MDT), calculated as $\begin{array}{c} {\mathrm{MDT}}_{jk}=\sum_{i=1}^{n_{t}}\omega_{i}n_{ijk}d_{i}/\sum_{i=1}^{n_{t}}\omega_{i}n_{ijk} \end{array}$, where $n_{t}$ is the total number of traps, $d_{i}$ is the distance from trap i to the release site of event *k*, and $n_{ijk}$ is the number of sterile males surveyed at trap i on day j after release event *k*; Maximum Distance Traveled (MaxDT); and Flight Range (FR). 

To estimate the FR, a linear regression was employed. The model regressed the log10 (annulus median distance) on the cumulative estimated recaptures performed at each recapture station (represented by the *x*-axis). The FR50 and FR90 represent flight distances. FR50 signifies the distance at which 50% of the individuals have achieved their maximum flight, while FR90 indicates the distance for 90% of the individuals. These values are derived from the regression equation. Specifically, they correspond to the *y*-axis values at 50% and 90%, respectively, of the maximum value on the *x*-axis. To estimate the diffusion coefficient (D) reflecting mosquito movement patterns, we employed Fick’s first law-based analysis of Mean Squared Displacement (MSD) [41] derived from capture distances of released sterile males.

The survival rate of sterile males was estimated by the linear corrected method [42,43] as follows: $\theta =\frac{e^{\alpha }}{N+e^{\alpha }}$, $s=\frac{e^{\beta }}{(1+{\theta )}^{1/d}}$, where α and β are the regression coefficients of the linear regression of the log-transformed captures as a function of time, *N* is the number of individuals released, θ is the recapture rate, d is the number of days after release, and s is the survival rate. The Probability of Daily Survival (PDS) in the field is estimated by regressing log10 (x + 1) of the number of recaptures against the day of recapture, where the antilog10 of the slope of the regression line is the PDS [44]. Mean life expectancy in the field (ALE) is calculated from the PDS as 1/−loge PDS.

We leveraged a modified Lincoln index incorporating small sample corrections and daily survival adjustments $P=\frac{\;[R\;*\;S\;*\;(n-m+1)]}{\;(m+1)}$ to estimate the wild male population size within the study area based on recapture data of released sterile males [45], where *R* is the number of originally marked males, *S* is the daily survival rate, *n* is the total number of recaptures of both marked and wild adult males, and *m* is the number of recaptured marked males. These data, together with the fertility rate of the eggs (hatched eggs and normally shaped eggs with the presence of embryo are considered fertile) collected in the release and control sites, allow us to estimate the sterile male competitiveness index under field conditions using the Fried competitiveness index based on observed fertility rates and the S/W male ratio [46].

At a given sterile-to-wild male ratio, $R_{sw}=M_{s}/M_{w}$, where $M_{s}$ is sterile males and $M_{w}$ is wild males in a homogeneously mixed population.

$$Fried\;index=\frac{H_{w}-H_{s}}{H_{s}-H_{rs}}/R_{sw}$$

where $H_{w}$ is the percentage egg hatch in the control site (i.e., the natural fertility); $H_{s}$ is the percentage egg hatch in the release site (observed fertility under a sterile-to-wild male ratio $R_{sw}$) and $H_{rs}$ is the residual fertility of sterile males. Using data from BGS, a nonparametric bootstrap approach [47] was applied to obtain a confidence interval for the estimate of the Fried index as described in Bouyer et al. [18].

## 3. Results

### 3.1. Weather Conditions in the Release and Control Areas during the MRR Study

The mean daily temperature in the District of Gampaha for the year 2021 ranged from 26.2 °C to 28.5 °C (mean = 27.4 °C, SD = 0.7, SE = 0.15). The mean (daily) temperature during the MRR study period in the District of Gampaha from January to February ranged from 26.4 °C to 27.10 °C (mean 26.75 °C). The mean daily relative humidity in the District of Gampaha for 2021 ranged from 87% to 74.2% (mean 81.4%, SD = 3.1, SE = 0.63). For the MRR study period of January–February 2021, the mean relative humidity ranged from 74.2 to 81.3% (mean = 77.75). No rainfall was recorded at the selected release and control sites during the MRR trial period (Supplementary Table S2).

### 3.2. MDT and Dispersal between Two Release Points 

Table 1 displays the MDT by the different colored males from two release points. The MDT from the first release point (pink and white) was greater than the MDT from the second release point. A Generalized Linear Model (GLM, df = 1, F value = 10.64, *p*-value = 0.0011) revealed a statistically significant difference in MDT between the two points, indicating that sterile males released from the first point traveled farther than those released from the second. We used the barycenter of the first two release points to estimate the overall values, assuming all release mosquitoes have the same color with different variability in terms of mean distance traveled. Later, we came up with the MTD formula to evaluate the overall values of the table (Table 1).

**Figure 2:**
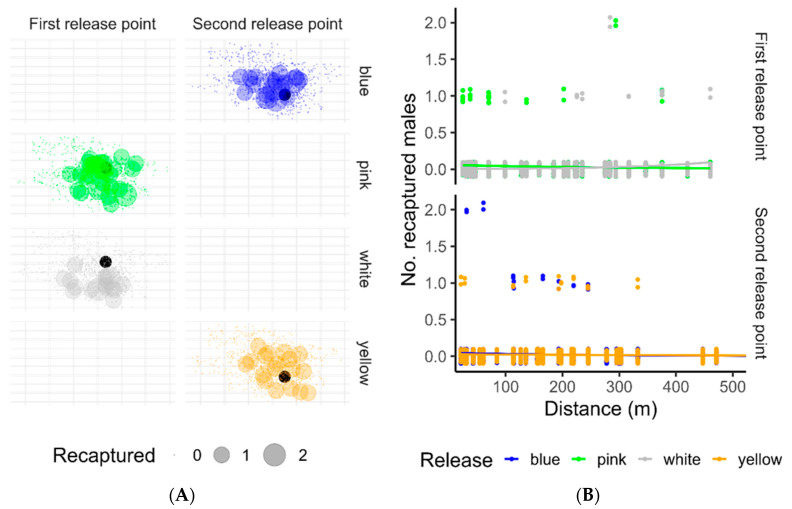
Distribution of accumulated recaptured sterile males in different collection stations (**A**) and dispersal pattern as a function of distance from the release point (**B**). The black dot in (**A**) represents the release point, while the size of the dots corresponds to the number of marked mosquitoes caught at each site.

Additionally, Figure 2 shows the recapture of sterile males as a function of distance from the release point. The dispersion pattern around the released point of the recaptured population remained similar between colors for each release series (Figure 2A). The number of recaptured marked males declined with distance from the release point (Figure 2B). It was observed that about 45.83% and 28.57% of sterile males were caught within 100 m, 73.24% and 65% within 200 m, and 90% and 80% within 250 m during the first and second releases, respectively.

### 3.3. Daily Survival and Mean Life Expectancy

Sterile males lived in the field up to 6 days after release with a mean life expectancy of a mean ± 3.55 ± 2.32 days (Table 2; Supplementary Table S3, Supplementary File S1).

### 3.4. Mating Competitiveness of Sterile Males Using Sit r-Package in Release Area

When the overall Fried index for mating competitiveness was calculated using selected parameters (BG and HLC) and the estimated value was 0.47 ± 0.007 (Table 3; Supplementary Table S4, Supplementary File S1).

### 3.5. Wild Male Population Size Estimation

Table 2 displays the estimated values of recapture data for BG sentinel traps and HLC. The Lincoln index estimated the mean population size to be 6988 and 2854 males in the overall estimated area of 30 ha for the first and second release series. The mean population size was 4921 males in the overall estimated area of 30 ha. Further, the mean wild male mosquito population density ranged from 95 to 232 males/ha (mean 163 males/ha).

### 3.6. Recapture Rates by Trap Type

The recapture rates of released sterile male mosquitoes were analyzed using three different trap types: BG-sentinel traps, HLC, and ovitraps. Ovitraps, primarily used to collect mosquito eggs, demonstrated the highest total and mean recapture rates, capturing 1787 eggs with a mean of 2.35 per trap and a standard deviation of 6.80. For adult mosquito collection, BG-sentinel traps and HLC were employed. BG-sentinel traps showed moderate recaptures with a total of 143 mosquitoes, a mean of 0.91 per trap, and a standard deviation of 1.28. HLC recorded the lowest recapture rates with 37 mosquitoes, a mean of 0.77 per trap, and a standard deviation of 1.15. These results indicate that while ovitraps are highly effective for monitoring mosquito egg deposition, BG-sentinel traps and HLC are essential for capturing and monitoring adult mosquitoes, with BG-sentinel traps being more effective than HLC.

## 4. Discussion

In the current study, MRR allowed us to determine the field competitiveness of sterile male *Ae. albopictus* mosquitoes as well as their dispersal and survival rates and the density of wild male mosquitoes in preparation for a future SIT pilot field trial in Sri Lanka to suppress a male *Ae. albopictus* mosquitoes. 

Four MRR releases during consecutive weeks were conducted during this study at two release points and mosquitoes were recaptured using BG traps and HLC. Suitable environmental weather conditions occurred during the study period, except for a few days. 

MDT and dispersal from two release points showed that all colored mosquitoes were collected beyond 230 m from their release point. Overall MDT was 255.1 ± 44.6 mm in the MRR test. Considering the MDT result in the current study, release points can be set up within 255 m distance from each other in the future pilot field trial site. Existing literature shows sterile male *Ae. albopictus* could disperse a mean distance of 93.85 ± 42.58 m and dispersed up to 258 m in MRR tests conducted using laboratory-reared males [7]. Studies conducted with wild populations of both males and females have reported mean mosquito travel distances exceeding 250 to 600 m [24,48,49]. In a recent review, the average estimated distance traveled by *Ae. aegypti* mosquitoes from the release site to the recapture site was 106 m [50]. Many studies suggest that wild *Ae. albopictus* are short-dispersing mosquitoes, generally moving fewer than a few hundred meters [28,51,52,53], which is in line with our results on sterile male dispersal under field conditions. Further, the recapture rate strongly depends on the dispersal capacity of the target species, on the recapture effort (density of recapture stations), and on the efficacy of the recapture method employed [54].

When we checked the survival of the sterile males in the field, we observed males surviving up to 6 days after release, with a mean life expectancy of 3.55 ± 2.32 days. With such a survival, one to two releases per week may be used within the planned suppression effort. In Albania, survival up to 15 days with a mean life expectancy of 4.26 ± 0.80 days was observed [7]. 

Results of the current study show efficient field mating competitiveness of sterile male mosquitoes based on Fried value 0.47 ± 0.007 and induced sterility in the wild eggs in the second generation. The Fried index value, or competitiveness index, is a measure of how competitive the sterile males are to mate with wild females. A value of 0 means that they are not able to compete at all with wild males, whereas a value of 1 means that they are equally competitive with the sterile males. A minimum value of 0.2 is necessary to conduct an efficient pilot field trial (18). Our current estimation of 0.47 ± 0.007 shows that the quality of the released mosquitoes was enough to conduct a future pilot SIT trial. In *Ae. albopictus*, the Fried competitiveness index was estimated at 0.28 in Albania (7), 0.39 (SD ± 0.75) in Italy (10), and 0.41 (95% CI 0.08–0.71) in Mauritius (11).

Mass production, manipulation, irradiation, and transportation procedures can affect the performance of sterile adult males in the field [23]. Therefore, the optimal conditions developed under the current study for mass production, manipulation, irradiation, and transportation can be applied for future pilot field trials planned to be conducted at the same site.

An estimation of the size of the wild mosquito population is necessary for the determination of the appropriate quantity of sterile males to be released for controlling the population effectively, which should be at least more than 10 times higher than that of the natural population size to see a quick suppression [19,31,55]. The current study showed a wild mosquito population density ranging from 95 to 232 males/ha (mean 163 males/ha), which shows a high density of wild mosquitoes. Considering the results of this study and other studies [19,31,55], release rates of more than 163 males/ha can be considered for future trials in Sri Lanka. The significant difference in wild male population sizes between the first and second release series could be attributed to several factors. One possible explanation is the natural variability in mosquito population dynamics, influenced by environmental factors such as temperature, humidity, and availability of breeding sites. Additionally, the timing of the releases and the effectiveness of the sterilization process may have varied between the two series, leading to differential impacts on the wild population sizes. 

When we compare our results with similar MRR studies conducted recently in other countries, we have medium density in the selected area. High population densities were reported in two cities in Italy 7000 (Caselle) and 3000 males/ha (Guisa Pepoli) in 2019 [23]. In a study conducted in southern Switzerland, 134 (Coldrerio) and 767 (Lugano) mosquitoes/ha were reported in 2018 [48]. 

Further, in our study, we estimated the PDS, mean life expectancy, and wild male population for two out of the four mosquito releases. This selective analysis was due to specific methodological and logistical constraints. Firstly, the two analyzed releases had complete and high-quality data sets that were enough recaptures necessary for accurate and reliable estimations. In contrast, the other two releases had lower recapture rates due to environmental factors and mechanical failure in some traps during the third release, which led to skewed data that could not be used for robust statistical analysis. Additionally, focusing on the two most reliable releases allowed for more accurate estimation, avoiding the introduction of bias from potentially flawed data. This approach ensures the accuracy and reliability of the results presented, providing a clear and valid representation of the PDS, mean life expectancy, and wild male population for the studied mosquito population. Future studies will aim to mitigate these issues to enable comprehensive analysis across all release events.

A sterile-to-wild male *Ae. albopictus* ratio of 5:1 was used in China for their pilot trial. The released mosquitoes (irradiated plus *Wolbachia* infected) at this ratio appeared to be able to induce high degrees of sterility [56]. An MRR trial conducted in Cuba with *Ae. aegypti* showed a density of 130.3 wild males/ha and a release rate of 800 to 1.600 sterile males/ha per week [17]. An operational SIT for a suppression trial conducted in Italy with *Ae. albopictus* showed a release rate of 896–1590 males/ha per week was effective in suppressing wild populations [9] with effective field competitiveness [10]. 

## 5. Conclusions

Based on the results of this study, as well as other studies on SIT for the control of *Aedes* mosquitoes [19,31,55], a release rate of more than 1630 sterile males/ha once a week with a 255.1 ± 44.6 m distance between two sterile male release points should be considered for future SIT trials in Sri Lanka. The data generated in this study on the distance between release sites, release frequency, and density will be useful for designing future *Ae. albopictus* suppression trials in Sri Lanka and other countries with similar situations.

## Figures and Tables

**Table 1:** Dispersal estimates of released sterile male mosquitoes for two-point MRR and population color.

MRR	Population	Distance Traveled (m)				
		MinDT	MaxDT	MDT	FR50	FR90
First release point	Pink	24.4	544.3	246.5	167.8	414.9
	White	24.4	544.3	331.7	236.8	505.4
Second release point	Yellow	21.2	460	211.0	150.0	353.8
	Blue	21.2	460	174.2	128.0	302.0
Overall		21.18	543.8	255.1	193.5	258.6

MinDT, minimum distance flown from the release point; MaxDT, maximum distance flown from the release point; MDT, mean distance traveled; FR50, flight range at 50% estimated recapture; FR90, flight range at 90% estimated recapture.

**Table 2:** Daily survival probability and the mean life expectancy of *Ae. albopictus* males in the field.

Releasing Series	Date 2021	Marked Sterile Males	No of Released Sterile Males	No of Dead Mosquitoes (Remained in the Cage)	Probability of Daily Survival (PDS)	Mean Life Expectancy (d)	Wild Male Population Size
First	1–22	Blue	5132	47	0.82	4.93	6988
		Pink	4956	59	0.82	4.93	
Second	1–29	White	5047	34	0.63	2.18	2854
		Yellow	4982	44	0.63	2.18	

PDS was calculated based on observations starting from the first release date (22 January 2021) for the first release series and from the second release date (29 January 2021) for the second release series. No of released sterile males refers to the actual number that was released and was able to fly out of the cage. No. of dead mosquitoes (remained in the cage) refers to the marked mosquitoes remaining in the release cage after 30 min of release. Dead mosquitoes were not considered in the statistical analysis.

**Table 3:** Mating competitiveness of sterile males.

Trapping Methods	BG-Sentinel Trap	HLC
Estimated Fred Index	0.46	0.45
S/W male ratio	0.47	0.49
Natural fertility	0.67	0.67
Observed fertility in SIT area	0.55	0.55
Sterile fertility (assumed)	0.01	0.01

BG-sentinel trap, HLC—Human Landing Collection.

## Data Availability

All data analyzed during this study are included in the published article. Further inquiries can be directed to the corresponding author.

## Supplementary Materials

The following supporting information can be downloaded at: https://www.mdpi.com/article/10.3390/insects15070466/s1, Annexure 1: Trap locations; Supplementary File S1: MRR Sri Lanka data analysis; Table S1: Chronology of sterile male releases in the release site; Table S2: Meteorological data in the District of Gampaha in 2021; Table S3: Adult survey; Table S4: Egg survey.
