# Supplementary material for: Mark–Release–Recapture (MRR) of Sterile Male Aedes albopictus (Skuse) in Sri Lanka: Field Performance of Sterile Males and Estimation of the Wild Mosquito Population Density
Source: Insects. 2024 Jun 22;15(7):466. doi: 10.3390/insects15070466 (PMC11276655; doi:10.3390/insects15070466)
Supplement: Supplementary file 1 [file insects-15-00466-s001.zip › Supplementary File S1-MRR_sri_lanka_data analysis-pdf.pdf]

```
library(lubridate)
library(sit)
library(sf)
library(tidyr)    # pivoting and reshaping
library(dplyr)    # selection and mutation
library(forcats)  # work with categorical variables (factors)


############################
date() # Current system time and date.
R.version.string # R version and version release date.
options(digits=5) # Confirm default digits.
options(scipen=999)# Suppress scientific notation.
options(width=60) # Confirm output width.

### Import data frame

rareal <- read.csv("rareal.csv", header=T,quote ="")

rpoints <- read.csv("rpoints.csv", h=T, dec=".")


traps <- read.csv("trap.csv", h=T, dec=".",sep=",",quote = "")

adult_sry <- read.csv("adult_survey.csv",header=T,
                        dec=".",sep=",",quote = "")

egg_sry <- read.csv("egg_survey.csv",header=T,
                  dec=".",sep=",",quote = "")


############################################################

### Release events
rareal_sit <- sit_revents(rareal)
rpoints_sf <- st_as_sf(rpoints,
                       coords = c("X_utm31","Y_utm31"),
                       crs = 4326)
rpoints_sit <- sit_revents(rpoints_sf)




traps_sf <- st_as_sf(traps,
                     coords = c("X_utm31","Y_utm31"),
                     crs = 4326)


ix_traps  <- sit_traps(traps_sf,
                       trap_types = sit_trap_types((data.frame(name = c("BG-
Sentinel","Human Landing Catch","Ovitrap"),
                                                               label = 
c("BGS","HLC","OVT"),
                                                               stage = 
c("adult","adult","egg")))))


#### data management
adult_sry <- adult_sry %>%
```

```
             mutate(date = as.POSIXct(interaction(Ed_year,Ed_month,Ed_day,sep = 
"-"),
                        format = "%Y-%m-%d"),
                    St_date = as.POSIXct(interaction(St_year,St_month,St_day,sep = 
"-"),
                                      format = "%Y-%m-%d"))%>%
             mutate(fwk = as.numeric(format(date,format="%W")))



alb_adult_data_clean <-
  adult_sry %>%
  ## Select and rename variables
  select(
    trap = Station.ID,
    type = Type.of.trap,
    start_date = St_date,
    survey = date,
    species = Collected.species,
    ## rename counts following a regular pattern "pop_sex"
    blue_male = No..Sterile.M.Blue,
    white_male = No..Sterile.M.White,
    pink_male = No..Sterile.M.Pink,
    yellow_male = No..Sterile.M.Yellow,
    wild_male = No..Wild.males,
    wild_female = No..Wild.females
  )


## Reshape into long format
adult_surveys  <-
  alb_adult_data_clean %>%
  pivot_longer(
    cols = blue_male:wild_female,
    names_to = "pop_sex",
    values_to = "n"
  )  %>%
  ## Separate variables
  separate(
    pop_sex,
    into = c("population", "sex"),
    sep = "_"
  ) %>%
  ## Include the missing duration of the sampling period
  mutate(
   duration =as.numeric(difftime(survey,start_date,units = "days"))
  )%>%
  select(trap,type,survey,species,population,sex,n,duration)




egg_sry <- egg_sry %>%
  mutate(date = as.POSIXct(interaction(Ed_year,Ed_month,Ed_day,sep = "-"),
                           format = "%Y-%m-%d"),
         St_date = as.POSIXct(interaction(St_year,St_month,St_day,sep = "-"),
                              format = "%Y-%m-%d"))%>%
  mutate(fwk = as.numeric(format(date,format="%W")))
```

```
alb_egg_data_clean <-
  egg_sry %>%
  select(
    trap = Station.ID,
    start_date = St_date,
    survey = date ,
    ##species = Specie,
    ## rename counts following a regular pattern "pop_sex"
    `TRUE` = No..Hatched.eggs,
    `FALSE` = No..not.Hatched.eggs
  )

## Reshape into long format
egg_surveys <-
  alb_egg_data_clean  %>%
  pivot_longer(
    cols = `TRUE`:`FALSE`,
    names_to = "fertile",
    names_transform = list(hatched = as.logical),
    values_to = "n"
  )  %>%
## Include the missing duration of the sampling period
mutate(duration = as.numeric(difftime(survey,start_date,units = "days")))%>%
  select(trap,survey,fertile,n,duration)



View(rareal)
rpoints
egg_surveys
adult_surveys


#
=================================================================================
#                               First Release
#
=================================================================================


# Target release point and reel point "2021-01-22"

f1_rareal <- subset(rareal,date == as.Date("2021-01-22"))%>%droplevels()
f1_rpoints <- subset(rpoints,date == as.Date("2021-01-22"))%>%droplevels()


# Target release date for adults survey
# first two weeks ("2021-01-25","2021-01-27")


f1_adult_surveys <- adult_surveys %>%
                    filter(survey == as.Date("2021-01-25") | survey ==
as.Date("2021-01-27"),
                    population =="blue" | population =="pink" | population
=="wild")%>%droplevels()

# Target release date for eggs survey first two weeks ("2021-01-29")
```

```
f1_egg_surveys <- subset(egg_surveys, survey == as.Date("2021-01-29"))%>
%droplevels()

#########

rareal_sit <- sit_revents(f1_rareal)
rpoints_sf <- st_as_sf(f1_rpoints,
                       coords = c("X_utm31","Y_utm31"),
                       crs = 4326)
rpoints_sit <- sit_revents(rpoints_sf)


ix_releases  <- c(rpoints_sit,rareal_sit)
ix_adults <- sit_adult_surveys(f1_adult_surveys)
ix_eggs <- sit_egg_surveys(f1_egg_surveys)




ix_sit <- sit(
  traps          = ix_traps,
  release_events = ix_releases,
  adult_surveys  = ix_adults,
  egg_surveys    = ix_eggs
)


print(ix_sit)
sit_competitiveness(ix_sit)
### SIT parameters to calculate
sit_mdt(ix_sit,by = "population")


sit_flight_range(ix_sit)
sit_flight_range(ix_sit, pool = TRUE)

sit_diffusion(ix_sit,spatial_adjustment = FALSE)
sit_diffusion(ix_sit, pool = TRUE)


sit_wild_size(ix_sit,pool = TRUE)

sit_survival(ix_sit)



#
==================================================================================
#                               Second Release
#
==================================================================================


# Target release point and reel point "2021-01-29"

s2_rareal <- subset(rareal,date == as.Date("2021-01-29"))%>%droplevels()
s2_rpoints <- subset(rpoints,date == as.Date("2021-01-29"))%>%droplevels()
```

```
# Target release date for adults survey
# weeks ("2021-02-1","2021-02-3")

#
s2_adult_surveys <- adult_surveys %>%
  filter(survey == as.Date("2021-02-1") | survey == as.Date("2021-02-3"),
         population =="white" | population =="yellow" | population =="wild")%>
%droplevels()

# Target release date for eggs survey first two weeks ("2021-02-5")

s2_egg_surveys <- subset(egg_surveys, survey == as.Date("2021-02-5"))%>
%droplevels()

#########

rareal_sit <- sit_revents(s2_rareal)
rpoints_sf <- st_as_sf(s2_rpoints,
                       coords = c("X_utm31","Y_utm31"),
                       crs = 4326)
rpoints_sit <- sit_revents(rpoints_sf)


ix_releases  <- c(rpoints_sit,rareal_sit)
ix_adults <- sit_adult_surveys(s2_adult_surveys)
ix_eggs <- sit_egg_surveys(s2_egg_surveys)




ix_sit <- sit(
  traps          = ix_traps,
  release_events = ix_releases,
  adult_surveys  = ix_adults,
  egg_surveys    = ix_eggs
)


print(ix_sit)
sit_competitiveness(ix_sit)
### SIT parameters to calculate
sit_mdt(ix_sit,by = "population")


sit_flight_range(ix_sit)
sit_flight_range(ix_sit, pool = TRUE)

sit_diffusion(ix_sit,spatial_adjustment = FALSE)
sit_diffusion(ix_sit, pool = TRUE)


sit_wild_size(ix_sit,pool = TRUE)

sit_survival(ix_sit)


#
==================================================================================
```

```
#                              Third Release
#
=================================================================================


# Target release point and reel point "2021-02-5"

s3_rareal <- subset(rareal,date == as.Date("2021-02-5"))%>%droplevels()
s3_rpoints <- subset(rpoints,date == as.Date("2021-02-5"))%>%droplevels()


# Target release date for adults survey
#
# survey == as.Date("2021-02-8") |

s3_adult_surveys <- adult_surveys %>%
  filter( survey == as.Date("2021-02-10"),
         population =="blue" | population =="pink" | population =="wild")%>
%droplevels()

# Target release date for eggs survey first two weeks ("2021-02-12")

s3_egg_surveys <- subset(egg_surveys, survey == as.Date("2021-02-12"))%>
%droplevels()

#########

rareal_sit <- sit_revents(s3_rareal)
rpoints_sf <- st_as_sf(s3_rpoints,
                       coords = c("X_utm31","Y_utm31"),
                       crs = 4326)
rpoints_sit <- sit_revents(rpoints_sf)


ix_releases  <- c(rpoints_sit,rareal_sit)
ix_adults <- sit_adult_surveys(s3_adult_surveys)
ix_eggs <- sit_egg_surveys(s3_egg_surveys)




ix_sit <- sit(
  traps          = ix_traps,
  release_events = ix_releases,
  adult_surveys  = ix_adults,
  egg_surveys    = ix_eggs
)


print(ix_sit)
sit_competitiveness(ix_sit)
### SIT parameters to calculate
sit_mdt(ix_sit,by = "population")


sit_flight_range(ix_sit)
sit_flight_range(ix_sit, pool = TRUE)

sit_diffusion(ix_sit,spatial_adjustment = FALSE)
```

```
sit_diffusion(ix_sit, pool = TRUE)


sit_wild_size(ix_sit,pool = TRUE)
sit_wild_size(ix_sit)

sit_survival(ix_sit)


#
================================================================================
#                              Forth Release
#
================================================================================


# Target release point and reel point "2021-02-12"

s4_rareal <- subset(rareal,date == as.Date("2021-02-12"))%>%droplevels()
s4_rpoints <- subset(rpoints,date == as.Date("2021-02-12"))%>%droplevels()


# Target release date for adults survey


###  survey == as.Date("2021-02-15") | survey == as.Date("2021-02-17") |

s4_adult_surveys <- adult_surveys %>%
  filter(survey == as.Date("2021-02-19") | survey == as.Date("2021-02-22") | survey
== as.Date("2021-02-24"),
         population =="white" | population =="yellow" | population =="wild")%>
%droplevels()

# Target release date for eggs survey first two weeks ("2021-02-19")

s4_egg_surveys <- subset(egg_surveys, survey == as.Date("2021-02-19"))%>
%droplevels()

#########

rareal_sit <- sit_revents(s4_rareal)
rpoints_sf <- st_as_sf(s4_rpoints,
                       coords = c("X_utm31","Y_utm31"),
                       crs = 4326)
rpoints_sit <- sit_revents(rpoints_sf)


ix_releases  <- c(rpoints_sit,rareal_sit)
ix_adults <- sit_adult_surveys(s4_adult_surveys)
ix_eggs <- sit_egg_surveys(s4_egg_surveys)




ix_sit <- sit(
  traps         = ix_traps,
  release_events = ix_releases,
  adult_surveys  = ix_adults,
  egg_surveys    = ix_eggs
```

```
)


print(ix_sit)
sit_competitiveness(ix_sit)
### SIT parameters to calculate
sit_mdt(ix_sit,by = "population")


sit_flight_range(ix_sit)
sit_flight_range(ix_sit, pool = TRUE)

sit_diffusion(ix_sit,spatial_adjustment = FALSE)
sit_diffusion(ix_sit, pool = TRUE)


sit_wild_size(ix_sit,pool = TRUE)

sit_survival(ix_sit)
```
