# Supplementary material for: Mark–Release–Recapture (MRR) of Sterile Male Aedes albopictus (Skuse) in Sri Lanka: Field Performance of Sterile Males and Estimation of the Wild Mosquito Population Density
Source: Insects. 2024 Jun 22;15(7):466. doi: 10.3390/insects15070466 (PMC11276655; doi:10.3390/insects15070466)
Supplement: Supplementary file 1 [file insects-15-00466-s001.zip › Supplementary Tables S1, S2.pdf]

Mark-Release-Recapture (MRR) of sterile male *Aedes albopictus* (Skuse) in Sri Lanka: Field performance of sterile males and estimation of the wild mosquito population density

Table S1. Chronology of sterile male releases in the release site

**Release series**	**Release point**	**Date 2021**	**Colour**	**No of released sterile male mosquitoes**	**No. of dead mosquitoes (remained in the cage)**	**Adult survey dates 2021**	**Egg survey dates**
First	First	01-22	Pink	4,956	59	01-25	01-29
	Second	01-22	Blue	5,132	47	01-27	
Second	First	01-29	White	5,047	34	02-01	02-05
	Second	01-29	Yellow	4,982	44	02-03	
Third	First	02-05	Pink	5,084	53	02-10	02-12
	Second	02-05	Blue	5,021	38		
Fourth	First	02-12	White	5,016	41	02-19	02-19
	Second	02-12	Yellow	4,975	49	02-22<br>02-24	

No of released sterile male mosquitoes refers to the actual number that were released and were able to fly out of the cage

No. of dead mosquitoes (remained in the cage) refers to the marked mosquitoes remaining in the release cage after 30 min of release. Dead mosquitoes were not considered in the statistical analysis

Table S2. Meteorological data in the District of Gampaha in 2021

Month 2021	Avg _Temp	Min_ temp	Max_ temp	Avg_ RH	Avg_ Rainfall	Avg_ rainy days	Ave_ WindSpeed
01	26.4	22.70	30.90	81.3	159.5	14	8.2
02	27.1	22.60	32.40	74.2	21.08	0	11.4
03	27.4	22.90	32.50	78.8	209.55	14	6.9
04	28.5	24.30	32.00	79.3	199.4	10	8.6
05	28.1	24.70	31.30	83.4	532.64	25	9.8
06	28.3	24.20	30.80	82.2	355.58	16	11.9
07	28.2	25.20	30.60	81.5	124.72	15	13.6
08	27.7	25.30	30.30	82.1	173.48	16	12.8
09	27.8	25.10	30.80	81.9	142.24	23	11.7
10	26.6	23.80	30.30	83.9	541.51	26	9.1
11	26.2	23.20	29.90	87	467.39	26	7.8
12	27	22.70	32.10	80.1	47.25	7	9
